# Evidence of integumentary scale diversity in the late Jurassic Sauropod *Diplodocus* sp. from the Mother’s Day Quarry, Montana

**DOI:** 10.7717/peerj.11202

**Published:** 2021-04-29

**Authors:** Tess Gallagher, Jason Poole, Jason P. Schein

**Affiliations:** 1 Biology Department, Union College, Schenectady, New York, United States; 2 Bighorn Basin Paleontological Institute, Red Lodge, Montana, United States

**Keywords:** Diplodocus, Dinosaur, Sauropod, Integument, Reptile, Fossil, Paleontology, Archosaur, Skin, Tubercle

## Abstract

The life appearance of dinosaurs is a hotly debated topic in the world of paleontology, especially when it comes to dinosaur integument. In the case of sauropods, however, the topic is harder to properly discuss due to the limited amount of fossilized skin impressions that have been discovered. Thus far, the fossil record of sauropod integument fossils include titanosaur embryos from Patagonia, possible keratinous diplodocid dorsal spines, track ways with foot impressions, and other isolated skin impressions found in association with sauropod body fossils. Several prominent integument fossils have been found at the Mother’s Day Quarry, located in the Bighorn Basin, Montana. These discoveries may bring new important information about diplodocids, specifically *Diplodocus* sp. Here we describe newly uncovered fossilized skin that gives evidence of scale diversity in the genus *Diplodocus*. The scales themselves represent tubercles, and exhibit various shapes including rectangular, ovoid, polygonal, and globular scales. The tubercles are small in size, the biggest of which only reach about 10mm in length. Considering how diverse the scale shapes are in such a small area of skin, it is possible that these distinct scale shapes may represent a transition on the body from one region to another: perhaps from the abdomen to dorsal side, or abdomen to shoulder. Based on analysis of extant integument and scale orientation of crocodilians, it is possible to hypothesize on the location of the integument relative to the body as well as the size and relative maturational status of the individual.

## Introduction

Life depictions of dinosaurs have changed considerably over time as a result of new discoveries and a better understanding of functional morphology. The most prominent example being the presence of feathers in several dinosaur clades (e.g., Xu et al., 2012), which has generated great interest in the evolution of birds and feathers, and ultimately changed how we view dinosaurs. However, our understanding of scaley dinosaur integument has also changed. Over the past century, the number of studies published on dinosaur scales has dramatically increased (e.g., Brown et al., 2017; Bell, 2014; Bell & Hendrickx, 2020). Despite this, research into sauropod integument remains rather limited compared to research on other dinosaur integument. Some of the best-preserved sauropod skin comes from titanosaur embryos in Patagonia (Coria & Chiappe, 2007), which show that these animals would have had diverse scale shapes and sizes as well as diverse patterns in terms of how the scales are oriented. Other information on sauropod skin is limited to footprints and skin impressions that show mosaic or pebble like patterning (Platt & Hasiotis, 2006; Del Valle Giménez, 2007; Kim et al., 2010; Foster & Hunt-Foster, 2011; Czerkas, 1994). Diplodocid integument fossils in particular are only known from several small skin impressions and carbon film fossils consisting of the patterns described above. The most noteworthy diplodocid integument discovered are dorsal spines that were found near the caudal region of a diplodocid fossil from the Morrison Formation, as this discovery sheds light on the potential scale diversity and appearance of diplodocids (Czerkas, 1992). New fossilized skin from *Diplodocus* sp. has recently been discovered at the Mother’s Day Quarry in Montana. Some of the first skin fossils discovered at this quarry exhibited polygonal scales (Myers & Storrs, 2007; Storrs, Oser & Aull, 2012). However, the skin fossils were primarily mentioned as evidence for taphonomic interpretations, and no in-depth description of the scale patterns and characteristics are available. In this paper, we describe newly discovered *Diplodocus* sp. carbonaceous skin from the Mother’s Day Quarry that consist of new scale shapes and patterns never before seen in diplodocids.

### Site background

The Mother’s Day Quarry, located in the Bighorn Basin, Montana, consists of Upper Jurassic deposits. The quarry has yielded over 2,500 fossils over the past two decades belonging to at least fifteen different *Diplodocus* individuals of a single indeterminate species (Myers & Storrs, 2007). Originally, the *Diplodocus* specimens were classified as “juveniles” and “subadults” due to their small size and unfused bones (Myers & Storrs, 2007; Storrs, Oser & Aull, 2012; Woodruff & Fowler, 2012). However, more recent analyses revealed that some of these individuals may be more histologically mature than previously thought, indicating there may be an additional dwarfed morphotype present in the Mother’s Day Quarry (Woodruff et al., 2018). Only two other taxa have been discovered at this site as represented by allosaur teeth and a single crustacean. The proposed reason this site contains mostly *Diplodocus* is that these individuals may have lived in a herd together, showing gregarious behavior (Myers & Fiorillo, 2009). Sedimentological and taphonomic evidence suggests that the *Diplodocus* skeletons are the result of a single mass mortality event, probably due to drought, followed by transportation and deposition in a high-density debris flow (Storrs, Oser & Aull, 2012).

### Skin preservation

Based on physical evidence, as well as prior studies of diplodocid integument as a carbonaceous film rather than an impression or mold. The preservation of the skin was most likely due to anoxic conditions caused by the rapid burial preventing the skin from decaying as evidenced by the presence of calcareous rinds on some of the long bones in the quarry (Myers & Storrs, 2007). In addition to anoxic conditions, the skin would have gone through a period of desiccation before being buried by the debris flow, which also aided in preservation as this would have toughened the skin and allowed it to survive for longer than other soft tissues (Storrs, Oser & Aull, 2012). The integument is preserved as a 3-dimensional relief and differs in coloration from the surrounding matrix, exhibiting grey, dark brown, or black coloration as opposed to the yellow and red of the surrounding siltstone. Some of the scale surfaces display a bumpy texture that can be described as multiple small “tubercles” within the scales. These small “tubercles” are most likely dermal papillae and are the result of degradation of the epidermis revealing the dermis underneath (Czerkas, 1994). Dermal papillae are a common presence in carbonaceous sauropod skin fossils (Czerkas, 1994; Foster & Hunt-Foster, 2011), but unlike other carbonaceous sauropod skin, the dermal papillae in this specimen are not as prominent and are only present on some scales. This could indicate that the epidermis is preserved in some areas of the fossil. The distinct coloration, presence of dermal papillae, and preservation in anoxic conditions gives evidence that this is the fossilized skin itself preserved as a carbonaceous film, rather than an impression.

## Materials and Methods

The fossilized skin, designated MDS-2019-028, is still *in situ* but excavation is planned for July 2021. The skin was found in proximity to two dorsal ribs also *in situ*: MDS-2019-009 and MDS-2019-010, though it is unknown whether the skin and ribs belonged to the same individual. Prior to excavation of the skin and rib fossils, photogrammetry will be used to create a digital replica.

**Figure 1:**
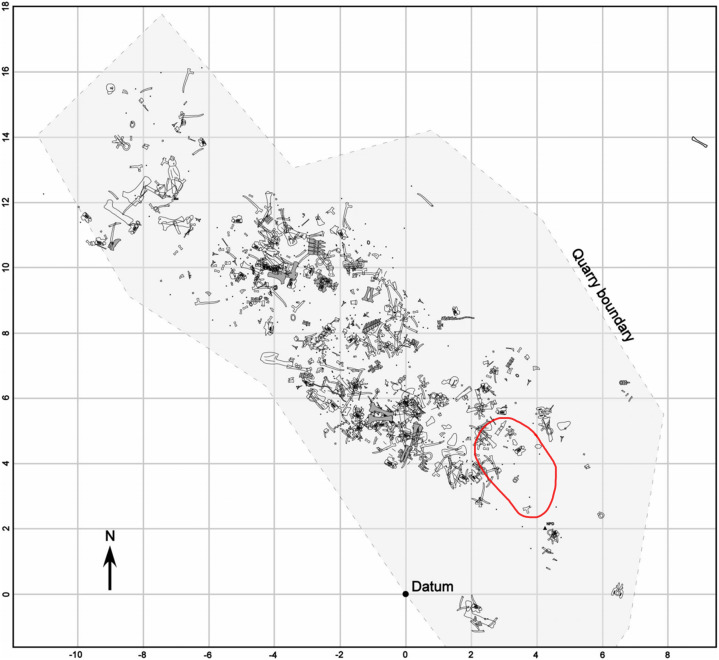
Quarry map of the Mother’s Day site showing bone location. Red circle indicates approximate location of skin and rib fossils. Quarry map modified from Myers & Storrs, 2007. Permission was granted from the Society of Sedimentary Geology for use of this figure.

The fossils will be prepared at the Academy of Natural Sciences in Philadelphia, and later stored at the Cincinnati Museum Center. Permits from the United States Department of the Interior Bureau of Land Management (permit numbers: MTM 109606, MTM 109606-e1, MTM 109606-e2, MTM 109606-e3, MTM 109606-e4) were issued to Schein et al. (2019) in order to allow for excavation of fossils. A quarry map has yet to be made but locations of all fossils have been recorded with a surveyor’s transit. Instead, we are utilizing an older quarry map to indicate the location of the skin and ribs (Fig. 1). All other pictures, drawings, and figures were created by Tess Gallagher.

**Figure 2:**
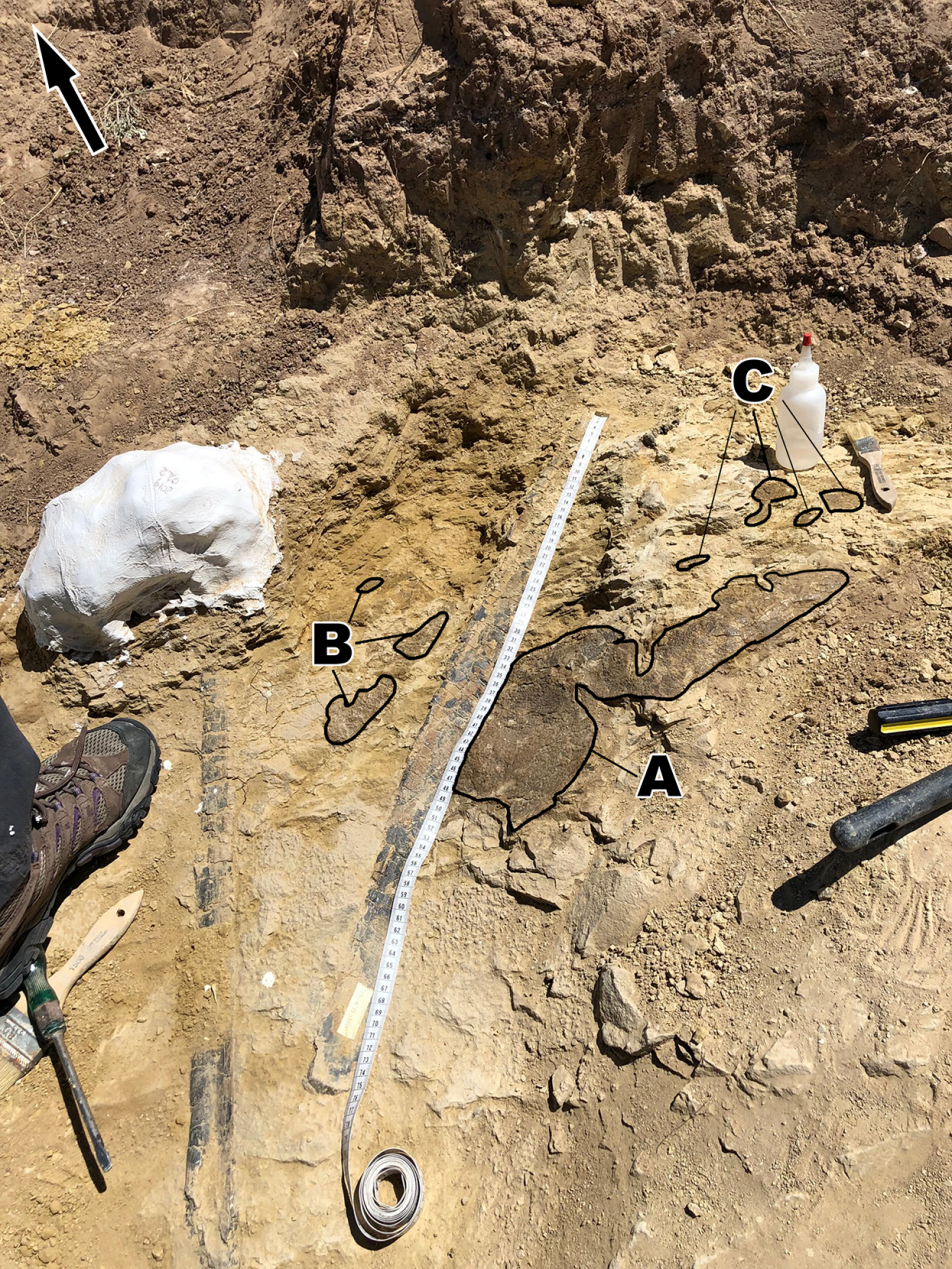
*Diplodocus* sp. skin in association with the two ribs. (A) the first and largest of the fragments found; showcases various scale shapes and patterns including the never before seen rectangular scales as well as the convex ovoid scales. (B) Skin fragments on the left side of the rib that most likely connect to (A). The fragment consists of tubercles of various shapes, the biggest of which are smooth in texture and are approximately ~10 mm in length. The other scales are smaller but vary in shape. They also appear to show a change in scale orientation. (C) Skin fragments that were most likely once connected to fragment A. These scales are located in a matrix of rock above (A). Scale shape and sizes include tiny ~2 mm tubercles and larger ~5 mm polygonal tubercles. The tape measure indicates the scale in centimeters. Arrow indicates North.

**Figure 3:**
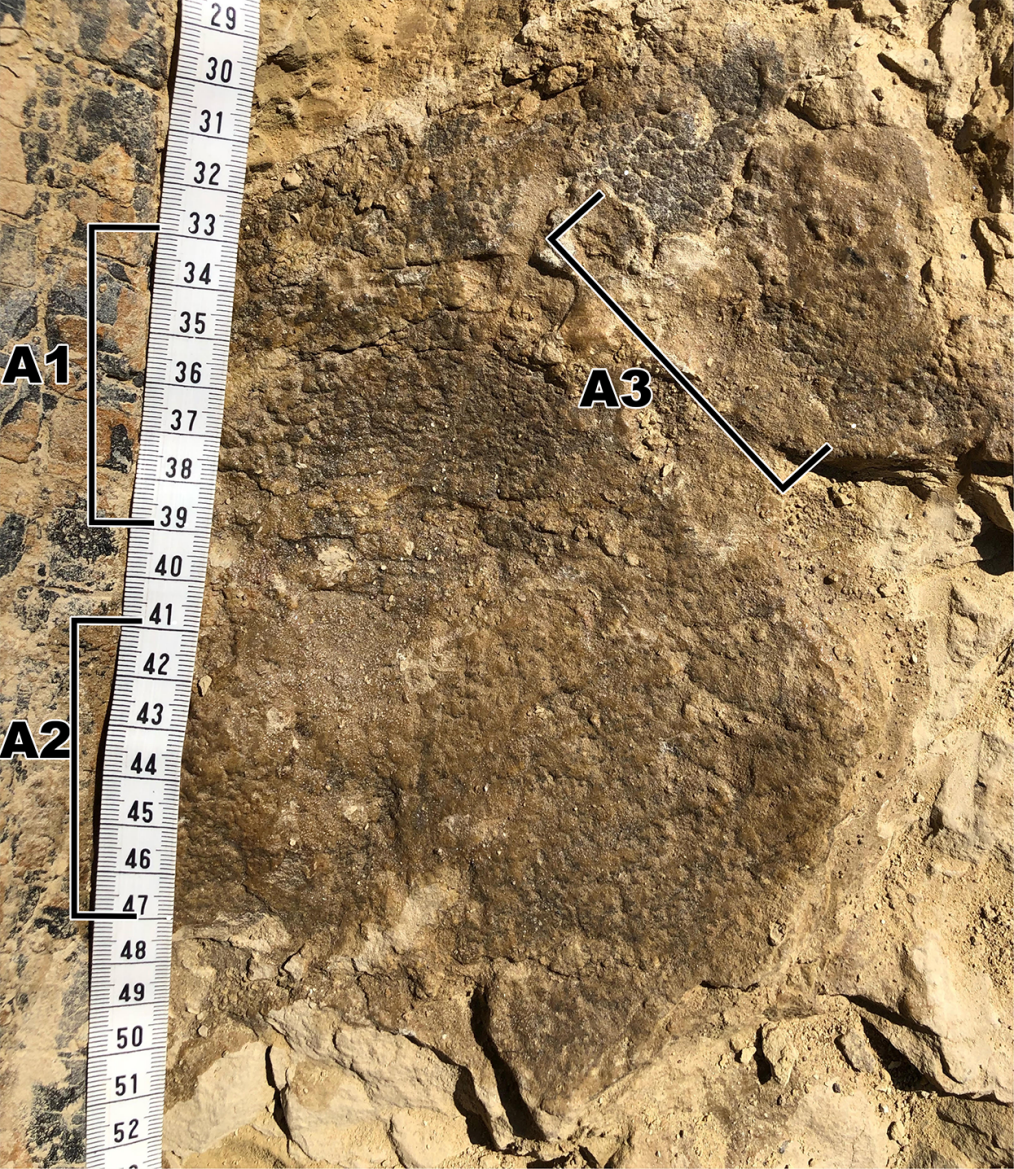
Close up of the largest area of skin fragment A with labeled sections of change in scale shape. (A1) Rectangular scales that range between ~5 mm to ~10 mm. (A2) Small polygonal scales that range in sizes of around ~5 mm as well as small pebble scales of about ~2 mm in size located to the left of the picture. (A3) Larger polygonal scales of similar size to the rectangular tubercles. Scale in cm.

To make description of the skin easier and to keep track of where the different scale shapes are in relation to one another, different areas of the skin have been designated as fragments identified with capital letters such as A, B, C (Fig. 2). On fragments A and B, sections of the skin that change in scale shape have been designated with numbers such as A1, A2, etc. Fragment C receives no such formatting since it lacks the scale diversity as seen on the other two fragments. Although it cannot be said with certainty where the skin belongs on the body, body area terminology will be used in quotation marks. Section A2 is designated as representing “ventral” (Fig. 3) while fragment B is designated as “anterior” (Fig. 2) to all other areas of the integument.

### Descriptions

The integument consists of non-overlapping scales, or tubercles, similar to those observed on other dinosaur skin fossils (e.g., Arbour et al., 2014; Bell, 2014; Christiansen & Tschopp, 2010; Czerkas, 1994; Czerkas & Czerkas, 1997; Kim et al., 2010). There is integument on both sides of the rib MDS-2019-010 (Fig. 1). The rib itself continues into the hill while the skin extends on the bedding plane surface. Although the skin and ribs were found in close proximity to each other, there are several variables that bring into question whether they belonged to the same individual. If the skin fossils belong to the same animal as the ribs, the skin should be preserved over the rib itself. Instead, it appears that the skin disappears underneath the rib. Furthermore the skin fossil has more scale diversity then would be expected in such a small area, especially when compared to the size of the individual from which the rib originated. Although it is not impossible for a *Diplodocus* of this individual’s size to have so much scale diversity, it would certainly be unexpected for reasons this discussed later in this paper. Despite the uncertainty of the relationship with the rib, the skin can still be attributed to the genus *Diplodocus*. One of the defining features of the Mother’s Day Quarry is the overwhelming abundance of *Diplodocus* material. Not only are there no other dinosaur taxa present other than the presence of *Allosaurus* teeth, which were most likely preserved from the result of scavenging, but even other non-dinosaur taxa such as turtles, crocodilians, etc. are completely absent from the quarry other than the presence of a single crustacean. Although it is possible there is non-*Diplodocus* fossil material at the quarry that has yet to be uncovered, *Diplodocus* would still be the most prominent taxon of the Mother’s Day Quarry, making *Diplodocus* sp. the most likely identity of the skin. The integument exhibits six different scale shapes, four of which are newly described for Diplodocus. The scale shapes are listed as follows:

1. *Polygonal*: the most common scale shape located in three different areas on the integument. Polygonal scales located “dorsally” are larger at 5 mm, while “ventral” polygonal scales are consistently <5 mm.
2. *Pebble*: the smallest of the scales at 1–2 mm, located “ventrally” alongside small polygonal scales.
3. *Rectangular*: scales that vary in length and overall size depending on placement, reaching between 2–10 mm. These scales are found abruptly cutting off the “ventral” polygonal scales, resulting in an abrupt change in scale shap as opposed to a more gradual change observed elsewhere.
4. *Globular*: irregularly shaped scales that lack defined angles or consistent patterning. They exhibit a more prominent 3-dimensional relief as compared to other scales except for the ovoid and dome scales. These scales measure 5–10 mm in diameter.
5. *Ovoid*: the consistently largest scales on the integument, measuring ∼10 mm in length. These scales are found clustered together, with the pointed ends of the ovoid pointing in the same direction, and also have a more prominent 3-dimensional relief than the rest of the scales. These scales also abruptly interrupt nearby polygonal scales.
6. *Dome*: two scales located several centimeters in front of the ovoid cluster of differing sizes. The larger scale is 5 mm while the smaller scale is <5 mm, and both exhibit prominent 3-dimensional relief similar to the ovoid scales.

The first and biggest fragment found measures about 240 mm in “dorsoventral” height and 600 mm in “anteroposterior” width (Fig. 2A). This skin appears to go underneath the rib, and might have originally been connected to fragment B, based on the similar scale size in both fragments. Fragment B is located “anterior” to fragment A (Fig. 2) and consists of three fragmented integument that range between 20–130 mm in “dorsoventral” height and 10–40 mm in “anteroposterior” width. Fragment C is located “dorsally” to fragment A and is the most “dorsally” located fragment. Fragment C consists of multiple small fragments that range in size from 20–100 mm in “dorsoventral” height and 20–50 mm in “anteroposterior” width. Considering how close fragment C is to fragment A as well as fragment C sharing similarly sized and shaped scales to fragment A, fragment C was most likely once connected to fragment A.

### Fragment A

**Figure 4:**
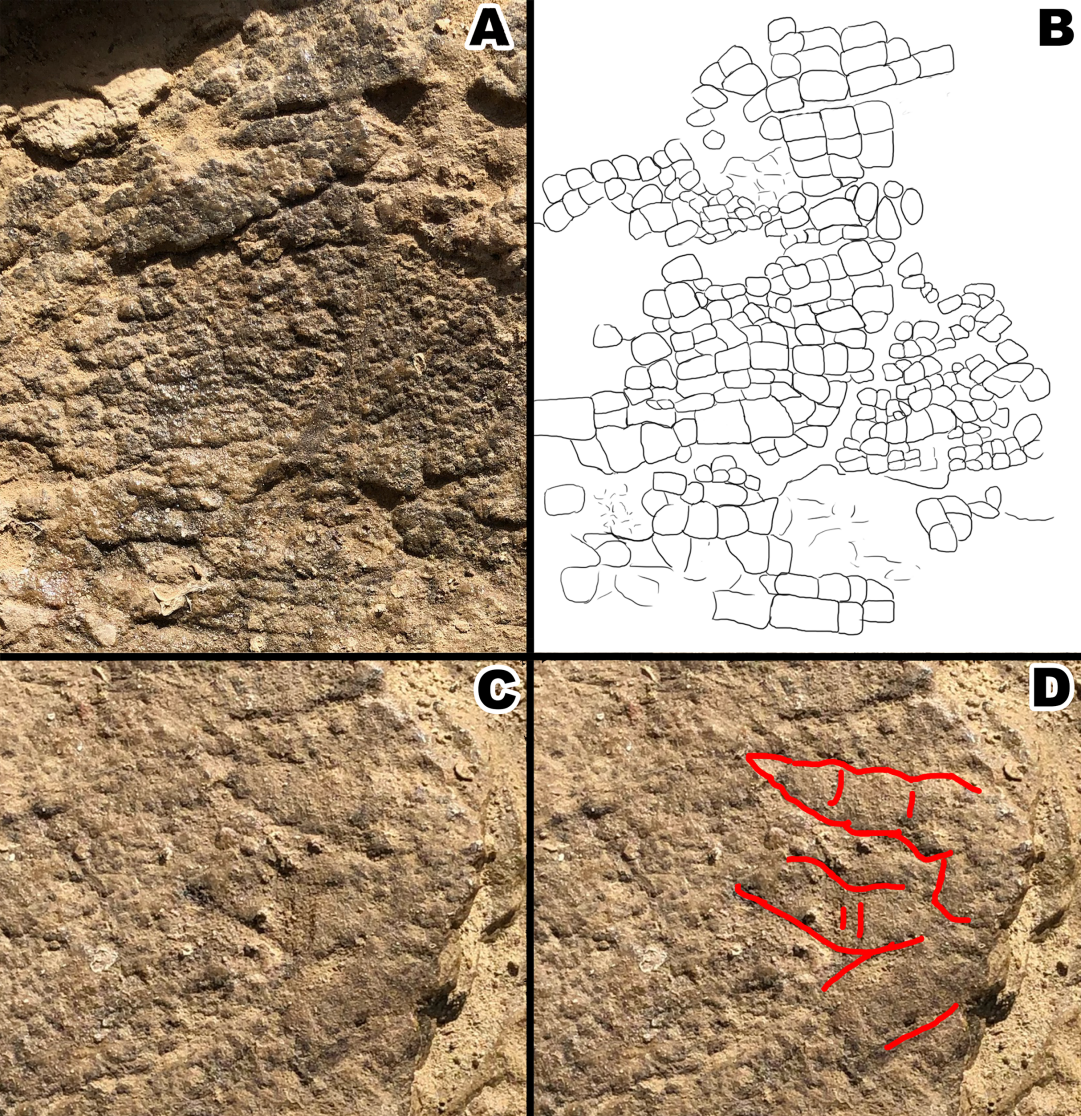
Section A1 exhibiting rectangular tubercles and possible footprint from section A2 with drawings for clarity. (A) Close up picture of section A1. (B) Interpretive drawing of section A1 to help distinguish individual rectangular scales. (C) Close up picture of possible footprint from section A2. (D) Interpretive drawing of footprint impression. Drawings by TG.

**Figure 5:**
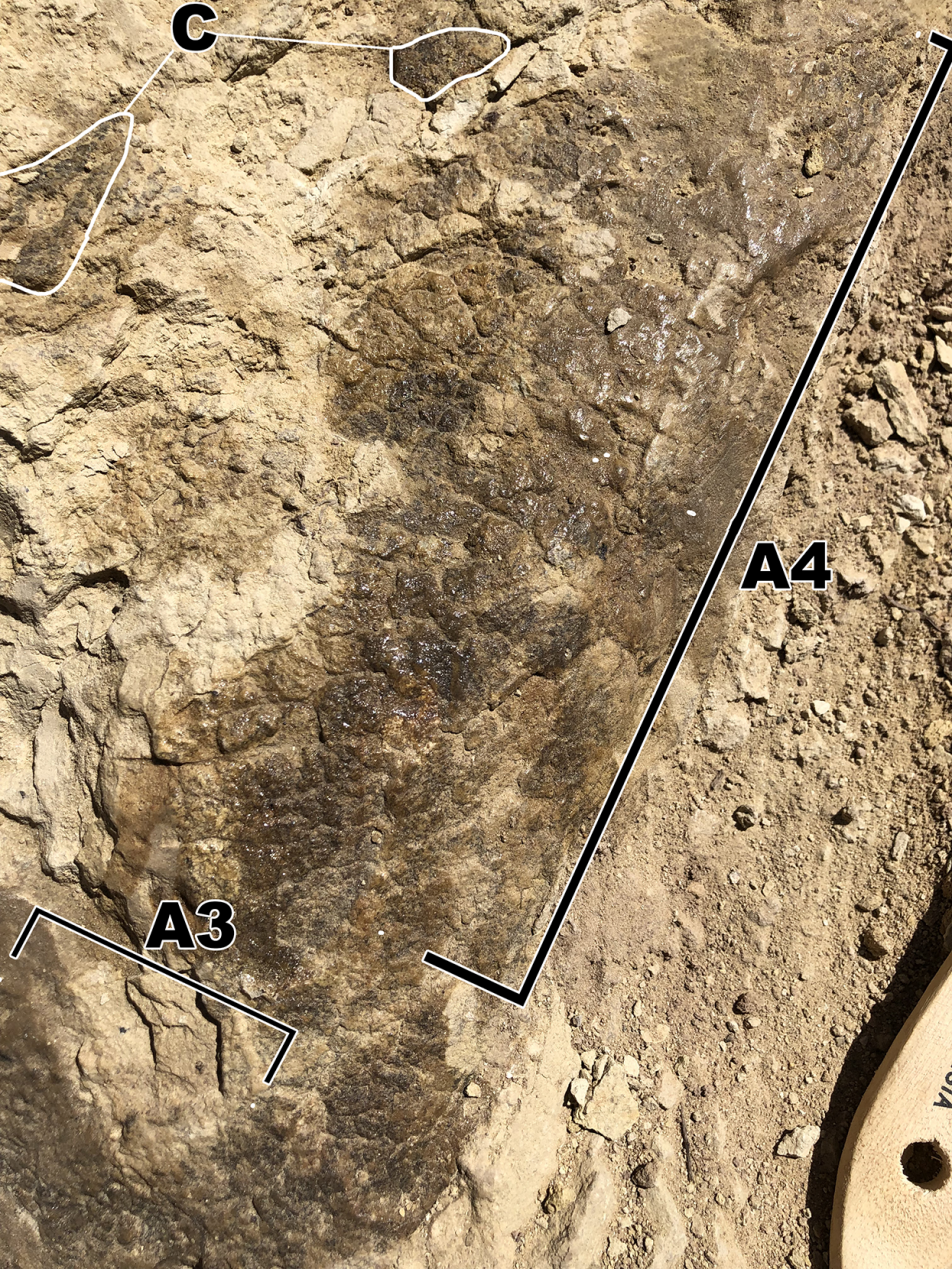
Close up picture of skin section A4 branching off from section A3 containing ovoid and dome scales. (A3) Polygonal tubercles. (A4) Polygonal scales of similar size to scales from A3, these then transition into the dome(<5 mm) and ovoid scales(~10 mm). (C) Pieces from fragment (C).

**Figure 6:**
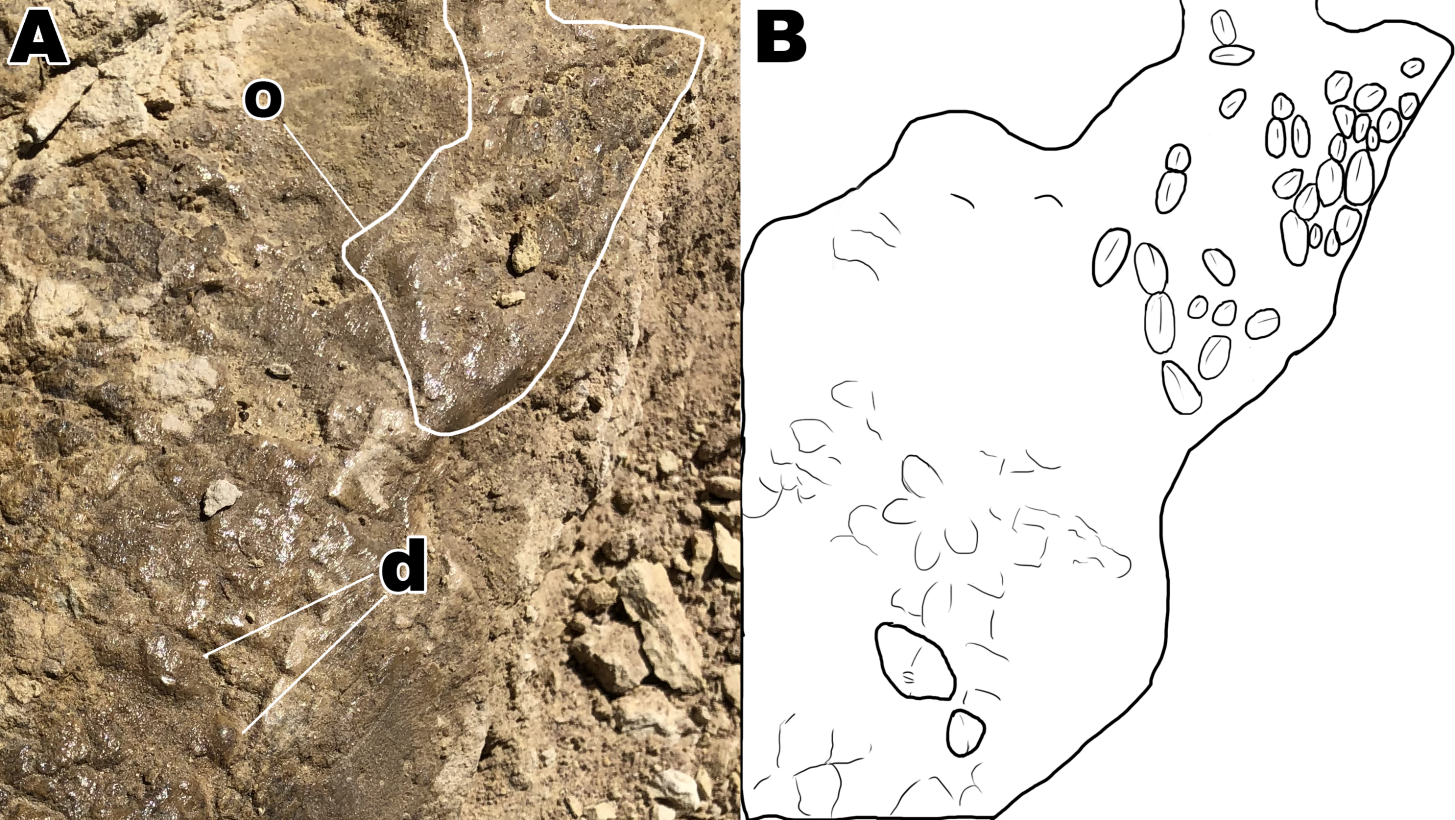
Close up of section A4 with a better view of unique scale shapes. (A) Dome scales and ovoid scales oriented in a cluster orientation. (B) Interpretive drawing to help highlight ovoid and dome scales from section A4. Abbreviations: d; dome scales, o; ovoid scales. Drawing by TG.

Fragment A contains signature pebble and polygonal scales on its lower region (designated A2 in Fig. 3) which measure less than 5 mm. These are similar in shape to scales observed in other diplodocid skin fossils as described by Czerkas (1992). To the right of section A2, the scales lose definition inside of two oblong shaped impressions in the skin itself that measure roughly 40 mm in length (Fig. 3). The current hypothesis is that this formation may represent a small dinosaur footprint, as this is the only area where the scales become non discernable, and the consistency of the oblong shapes mimic the look of other known dinosaur footprints, with what could be interpreted as individual toe pads present. However, it is also possible this formation could have been caused by other taphonomic processes, such as wrinkling of the integument created during burial. If these impressions do represent a footprint, the footprint would have most likely been made while the skin was desiccating, before it was buried in a debris flow. To the farthest left of section A2, the scales are even smaller in size (~1 mm) and may correspond to the small scales in section B. The scales change shape from small pebble and polygonal scales at the lower region of A2 into rectangular scales in the upper region designated A1. In section A1, rectangular scales are visible ranging in size from ~5 mm to ~10 mm (Fig. 3). The scales in section A1 change from minuscule ~1 mm scales to the larger rectangle scales from “posterior” to “anterior” (see Figs. 3 and 4) and change into polygonal scales “dorsally” (Fig. 4). Rectangular scales have been observed before in sauropods, most notably in embryonic titanosaur scales from Patagonia (Coria & Chiappe, 2007). However, the rectangular scales of the titanosaur embryos are neatly lined up and overlap each other. In addition, these rectangular scales observed in the Patagonian embryo are much larger than the surrounding scales. The rectangular scales on the *Diplodocus* specimen instead do not display such a specific pattern, showing multiple rows of straightly aligned rectangular and square scales. These rectangular scales then transition into more polygonal scales in section A3 that are around 5 mm in diameter. The polygonal scales transition into smooth ovoid scales in section A4 measuring ~10 mm in length and are also more raised than the other surrounding scales (Fig. 5). These scales are closely clustered together, and all oriented similarly; the pointed ends of the scales pointing towards section A3. This cluster measures roughly 30 mm by 70 mm. Between the cluster of ovoid scales and the polygonal scales of section A3, there are two smaller scales that are slightly raised and smooth in texture but are instead domed rather than ovoid (Fig. 6). They are located a few centimeters in front of the ovoid scale cluster, exhibiting no clear organized scale pattern. In addition to the dense cluster of ovoid tubercles, section A4 also displays another curious arrangement of scales. At the forefront of the cluster of ovoid scales, where the ovoid scales meet the polygonal scales, there is an arrangement of five ovoid scales in an arrow-like shape pointing towards section A3. The arrow orientation consists of a single scale at the point and two scales on each side. The ovoid scales look similar in nature and orientation to scales seen dorsally on modern day reptiles. Also taking into consideration the possible existence of dorsal spines on diplodocids (Czerkas, 1992), these ovoid scales may be homologous and may have also been present on the dorsal side of the animal, though whether these ovoid scales would have eventually grown into dorsal spines or kept their shape throughout life is up to debate.

### Fragment B

**Figure 7:**
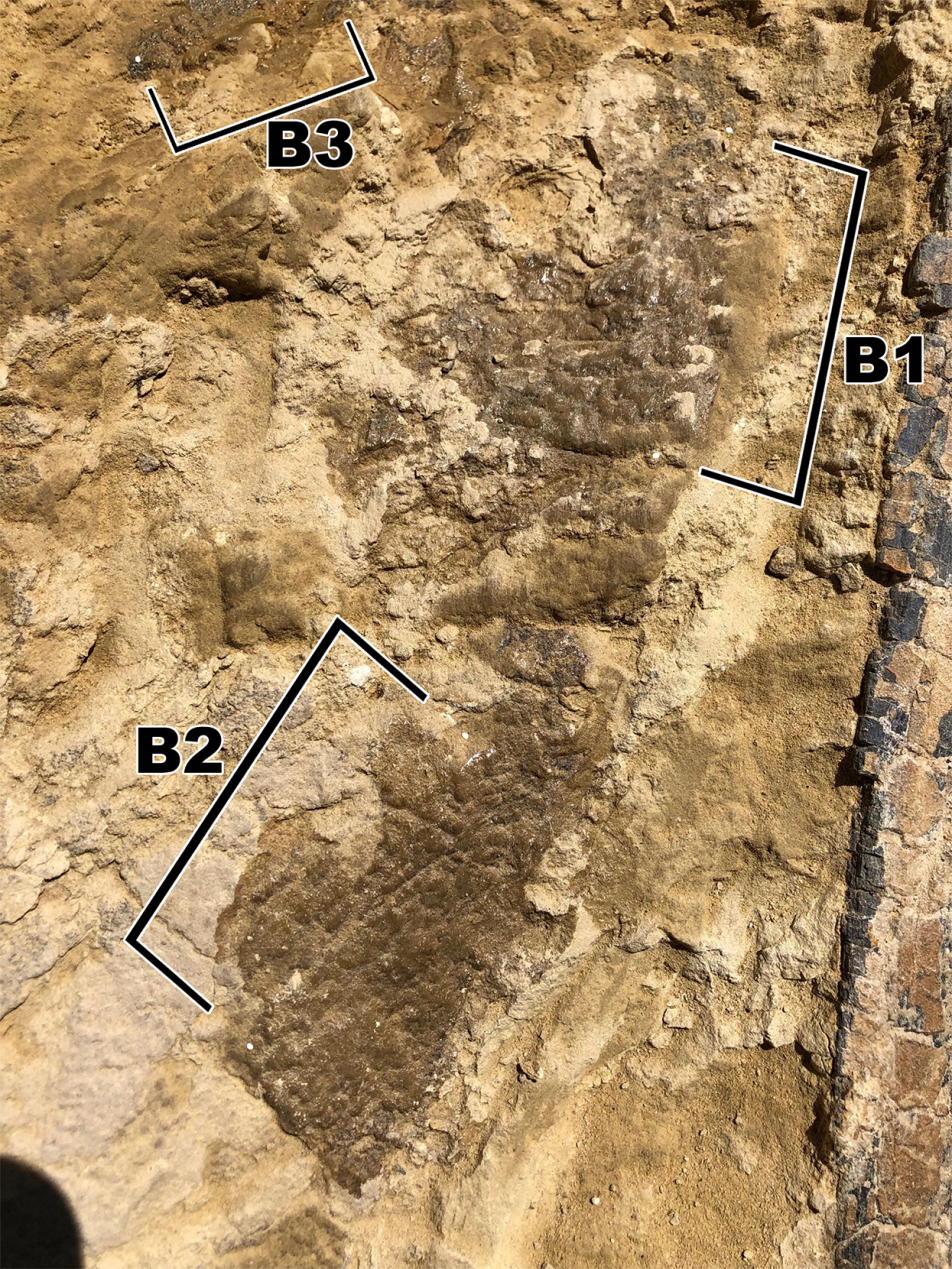
Skin fragment B, located on the opposite side of the rib as to fragment A. (B1) Smooth globular scales that measure ~10 mm. (B2) Polygonal and square scales that measure <5 mm. (B3) Pebble scales that measure ~2 mm.

**Figure 8:**
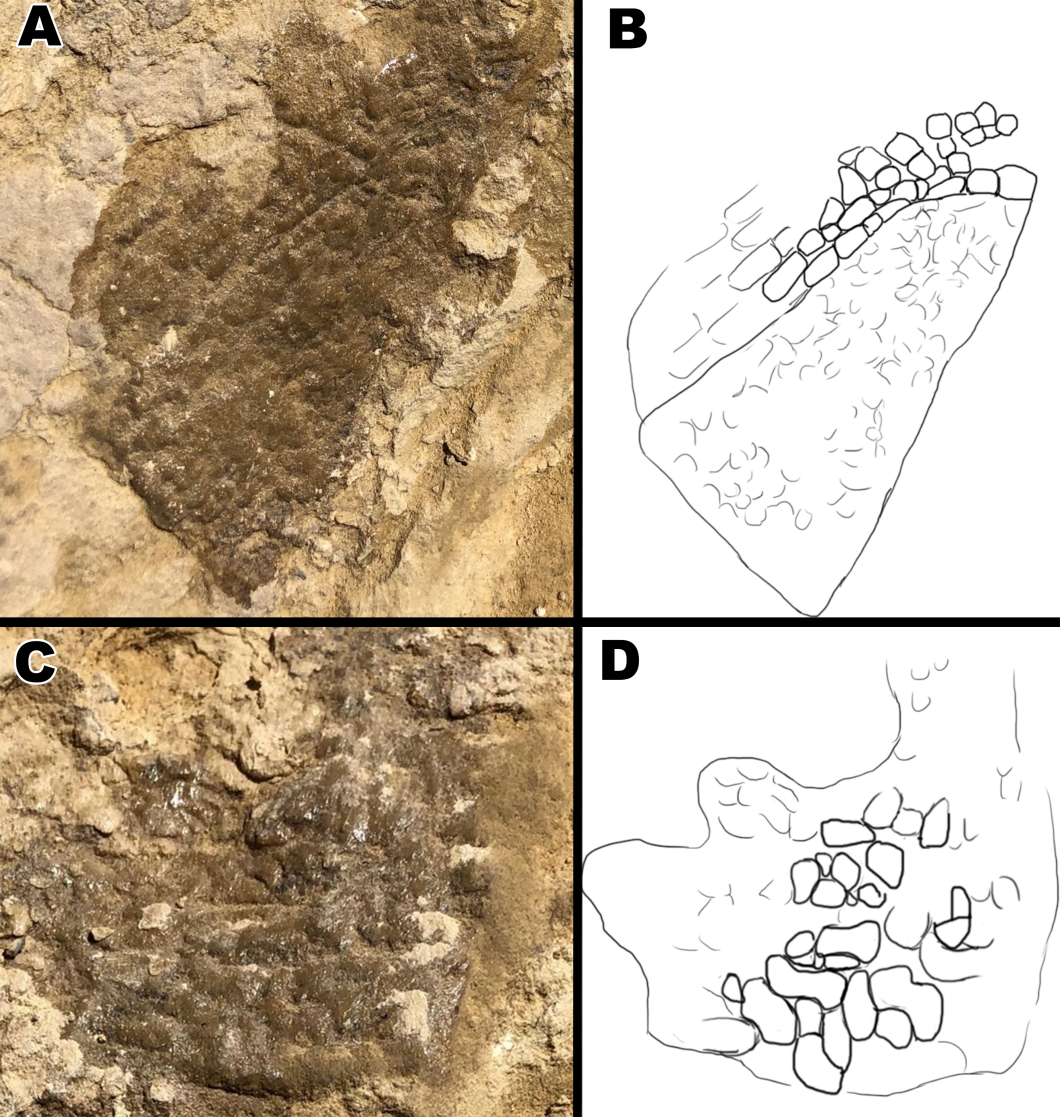
Close up pictures of section B1 and B2 for better view of the globular scales and arching orientation with interpretive drawings. (A) Close up of section B2. (B) Drawing of arching scale alignment from section B2. (C) Close up picture of section B1. (D) Drawing of globular scales from section B1. Drawings by TG.

**Figure 9:**
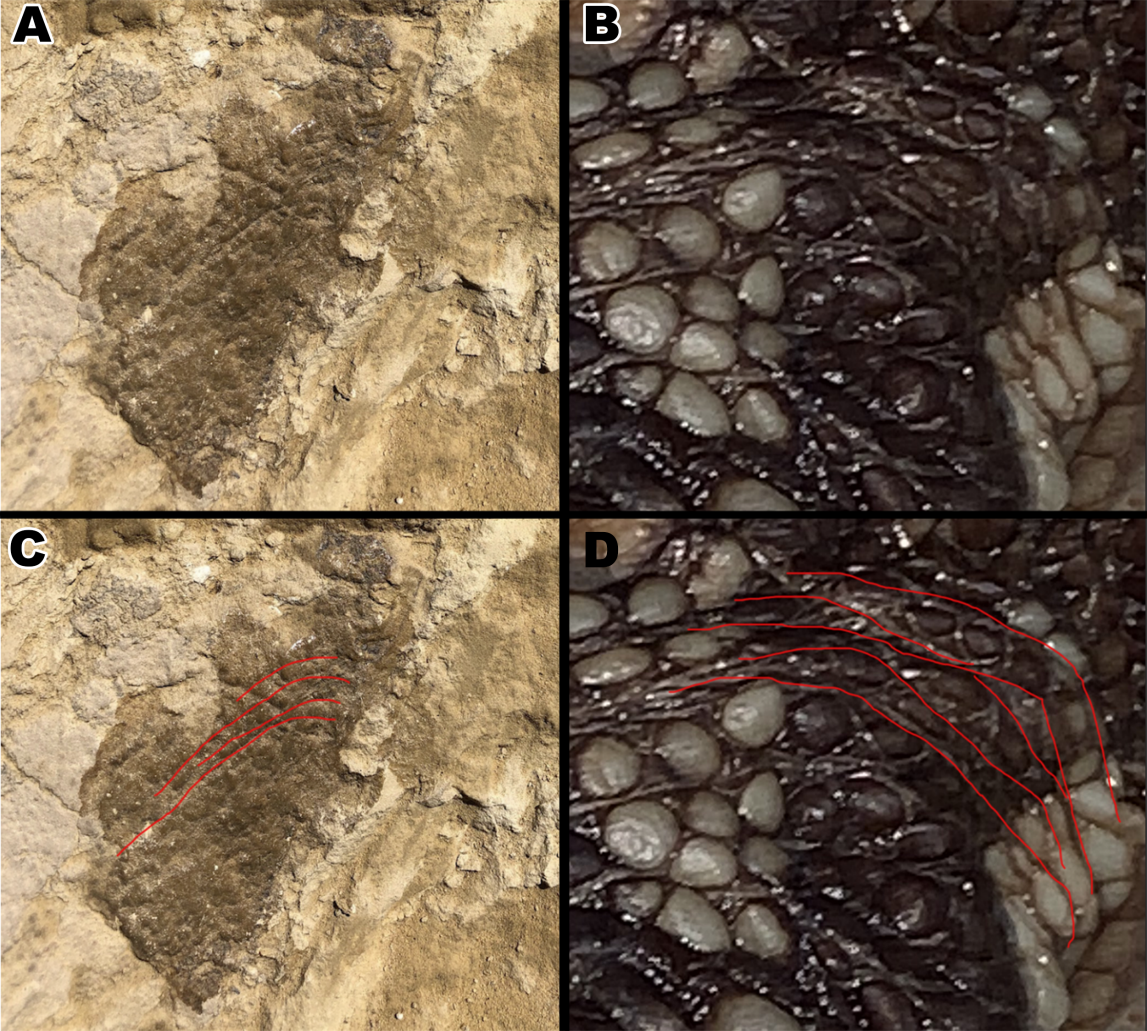
Comparison between scales around an *Alligator mississippiensis* hindlimb to downward aligned *Diplodocus* scales. (A) Close up picture of section B2. (B) Hindlimb of a juvenile Alligator with scales arching around where the back leg connects to the body. (C ) Arching scale rows of B2 outlined by red lines. (D) Arching scale rows of juvenile Alligator outlined with red lines.

Skin fragment B (Fig. 7) shows similar sized scales to fragment A but exhibits a different scale orientation “ventrally” as well as a different scale shape “dorsally”. Scales in fragment B1 are similar in size to those observed in fragment A1 but are irregular in shape with bean and globular-shaped tubercles arranged in a puzzle-like formation, often seen ’hugging’ or folding over nearby tubercles of similar shape (Fig. 8). The scales also display more rounded edges compared to the tubercles observed in fragment A, and have deeper, more visible indentations in-between each scale. Section B2 consists of square and polygonal scales, with sizes comparable to A2. An interesting feature in section B2 is that the small square scales are organized in linear rows that arch downwards, interrupting the nearby polygonal scale patterning (Fig. 8). There are at least two additional rows of arching scales next to the row closest to the polygonal scales. This patterning is very similar to scale patterning seen around crocodilian limbs (Fig. 9), which may suggest that this section may have been from a limb region in life. Skin section B3 consists of small <5mm pebble-like scales.

### Fragment C

**Figure 10:**
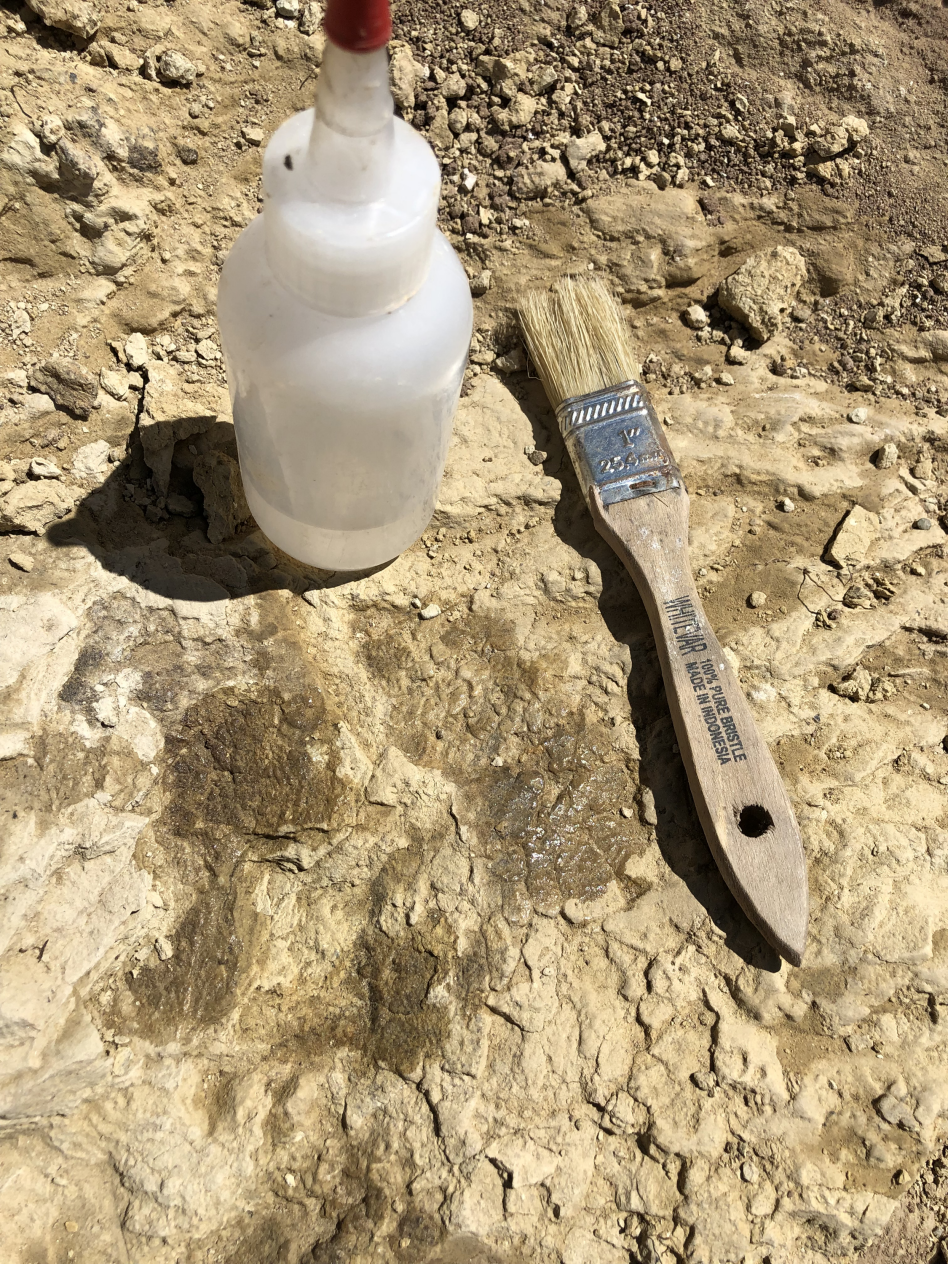
Skin fragment C with brush for size reference. These tubercles are within close proximity to section A4, and show similar polygonal patterning.

Fragment C (Fig. 10) consists of multiple small pieces of skin. The scales range in size from 2 mm to 5 mm. The scales appear to change in size depending upon their location: fragments closer to section A4 are smaller than those closest to A3. The fragments exhibit the same polygonal scales seen on section A3 and are also close in proximity and lay on the same bedding plane, so it can be assumed that these fragments were at one point attached to fragment A.

## Discussion

Through close examination of the integument, the evidence suggests that the skin belonged to a small individual, possibly of “juvenile” or even infantile maturation. The evidence we used to come to this conclusion is the presence of small, potentially “juvenile” bones in the Mother’s Day Quarry, the significant diversity of scale shapes over a small area of the integument, the small size of the scales, and the orientation of the scales implying the presence of a small limb. Each of these pieces of evidence is further discussed below.

**Figure 11:**
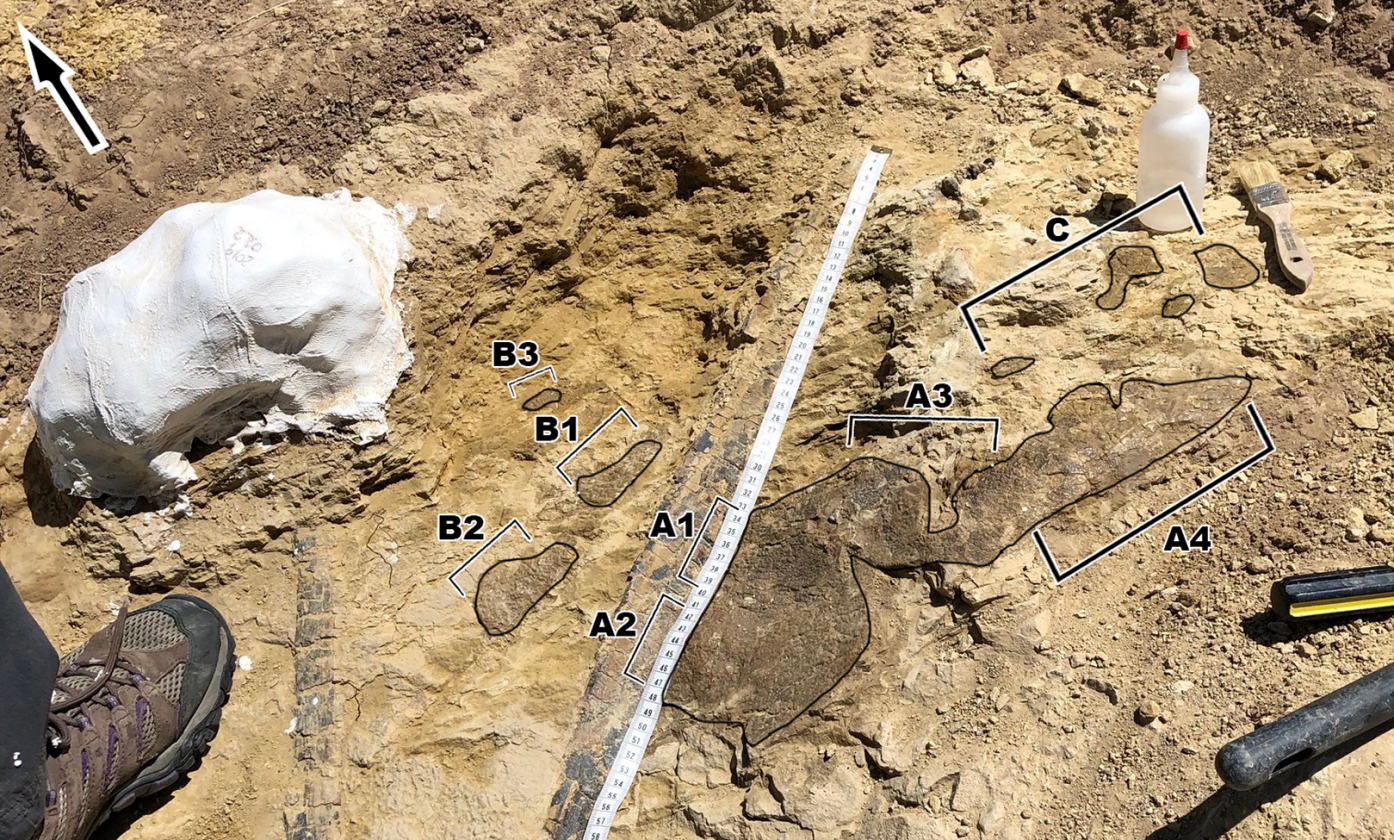
Picture of MDS-2019-028 showing placement of all sections of the integument. Fragment A exhibits the greatest amount of scale diversity while fragment C exhibits the least amount. Arrow indicates North.

**Figure 12:**
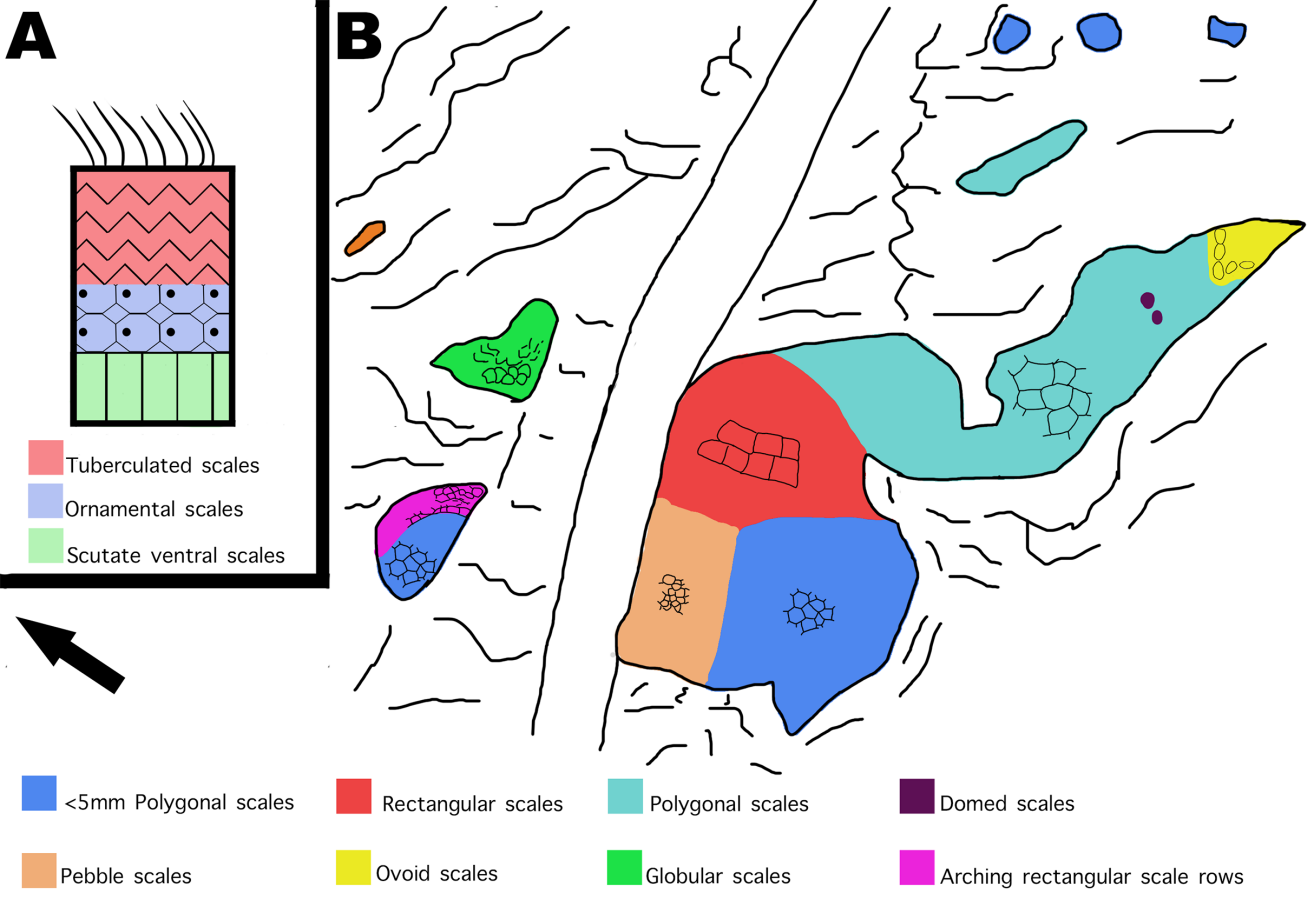
Distribution of scale shapes and orientations on MDS-2019-028 compared to scale shape distribution in *Juravenator*. (A) Simplified drawing of distribution of scale shapes in the tail of *Juravenator*. Different scale shapes are indicated by color and simplified drawings of the scales. Colored squares indicate what color is associated with each scale shape. Drawing based off descriptions from Bell & Hendrickx (2020). (B) Drawing of MDS-2019-028 showing distribution of scale shape and orientation. Change in scale shape or orientation is indicated with color, as indicated by colored squares on the bottom. Drawings by TG. Arrow indicates North.

**Figure 13:**
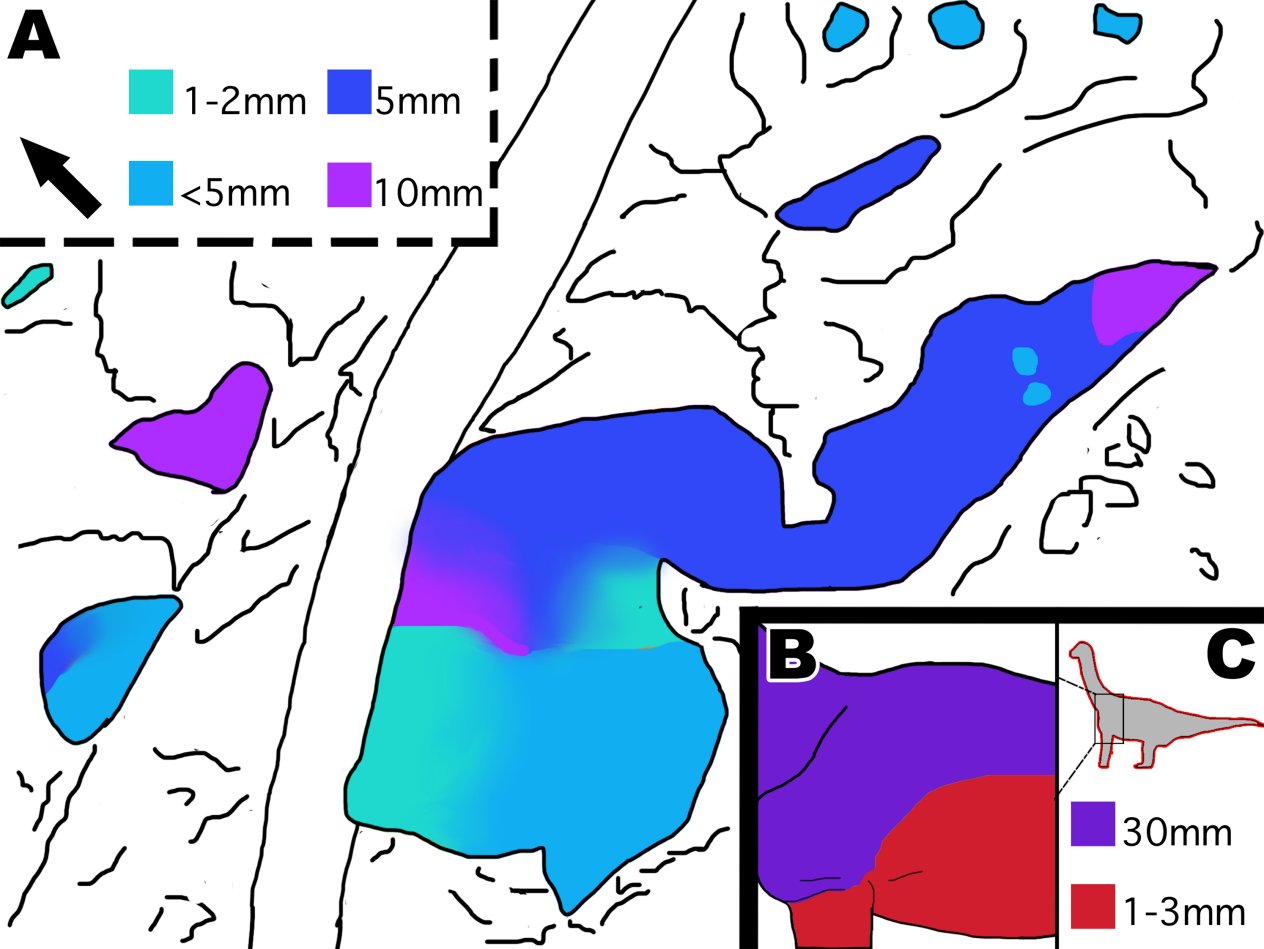
Distribution of scale size indicated by color in MDS-2019-028 and *Tehuelchesaurus*. (A) Interpretive drawing of distribution of scale size in MDS-2019-028 indicated by color. Color grading represents gradual transition in scale size, while abrupt color change indicates abrupt change in scale size. Squares indicate what color represents each scale size in mm. (B) Interpretive drawing of distribution of scale sizes in *Tehuelchesaurus* ventrally and dorsally. (C) Location of scales on the body and colored squares representing scale sizes in mm. It should be noted that it is unknown if *Tehuelchesaurus* had more diversity in scale size then what is known, and that the drawings are based off the idea that big scales were located dorsally and small scales located ventrally. Drawing of *Tehuelchesaurus* scale placement based off description from Del Valle Giménez (2007). Drawings by TG. Arrow indicates North.

Young and small individuals have been reported from this quarry, which range between 38–75% the size of known adult Diplodocus specimens. Woodruff et al. (2018) even reported to have found the smallest *Diplodocus* skull ever uncovered. Therefore, it is not unexpected that skin fossils found in the same bonebed are from a small and potentially young individual. However, it should be noted that there is still much debate on the maturational status of the Mother’s Day Quarry *Diplodocus*. Although there is evidence present at the Mother’s Day Quarry that the *Diplodocus* were “juvenile”, some of the elements used to determine maturational status have been brought into question. For instance, increase in neural spine bifurcation was originally thought to be a key characteristic in diplodocid ontogeny (Woodruff & Fowler, 2012). However, other studies have shown that variation in sauropod vertebra can mimic ontogenetic features and are thus not reliable in determining maturational status (Wedel & Taylor, 2013). Although we do believe the fossilized skin belongs to a small and potentially “juvenile” individual, we must stress that there is still much debate surrounding the maturational status of the Mother’s Day Quarry *Diplodocus*, and that more research is required in order to determine if the individuals are of adult or juvenile status. The integument represents a relatively small area in comparison to the overall body size of what would be an adult *Diplodocus* sp. Despite this, the integument shows a significant diversity of scale shapes and orientations (Fig. 11) especially when compared to other known Morrison diplodocid integument fossils. All other examples of diplodocid integument from the Morrison formation, except the possible *Kaatedocus* dorsal spines, have only ever exhibited polygonal or hexagonal scales (Czerkas, 1994; Foster & Hunt-Foster, 2011; Myers & Storrs, 2007). Change in scale shape across integument is usually indicative of a transition from one body part to another, as evidenced by modern reptiles and other dinosaur taxa. The theropod *Juraventator* exhibits three distinct scale shapes on its tail, of which the scale shape varies based on whether it is dorsally or ventrally located. Specifically, the scale shapes change ventral to dorsal in the following order: scutate ventral scales, ornamental scales, tuberculate scales, and finally feathers at the most dorsal part of the tail (Bell & Hendrickx, 2020). MDS-2019-028 shows a similar transition, featuring up to six different scale shapes localized on different areas of the integument that change in a “dorsoventral” and “anteroposterior” manner (Fig. 12). Such scale diversity has also been documented in the past for sauropods, specifically in titanosaur embryos. The skin of these titanosaur embryos exhibits six different scale arrangements consisting of various scale shapes, including parallel rows of large tubercles, striatelike rows, etc. (Coria & Chiappe, 2007). Although exact location of these scale arrangements on the body is unknown, the diversity in scale shapes and orientations on these specimens are indicative of differing areas of placement on the body. The reason why these embryos have such scale diversity preserved is most likely due to their size. Specifically, a greater area of integument relative to the body is more likely to preserve in a smaller sauropod versus a large, full-grown sauropod. This could be the reason for why MDS-2019-028 expresses more scale diversity compared to other diplodocid integument fossils. In addition to the diverse orientation of the scales, the polygonal scales described on MDS-2019-028 are much smaller than other known diplodocid polygonal scale fossils. For example, the biggest polygonal scales found during the reopening of the Howe Quarry in 1990 reach 30 mm in size (Czerkas, 1994; Tschopp, Mehling & Norell, 2020), apatosaurine diplodocid polygonal scales from Mygatt-Moore Quarry have an average diameter of 25 mm (Foster & Hunt-Foster, 2011), and previously discovered *Diplodocus* sp. polygonal scales from the Mother’s Day Quarry measure 10 mm (Myers & Storrs, 2007). None of the polygonal scales observed in MDS-2019-028 exceed 5 mm in size, making the biggest of these polygonal scales six times smaller than the biggest recorded diplodocid polygonal scales and half the size of other Mother’s Day polygonal scales. Furthermore, the Mygatt-Moore apatosaurine scales were found in association with large *Apatosaurus* material, which indicates that 30 mm polygonal scales are a distinctive trait of larger sized diplodocids. In terms of both scale size and orientation, another comparison can be made to the fossilized skin of the non-diplodocid sauropod *Tehuelchesaurus*. The polygonal scales of *Tehuelchesaurus* greatly vary in size measuring between 1 mm to 30 mm in diameter (Del Valle Giménez, 2007). Although the presence of small scales on a big sized sauropod may bring into question the reliability of comparing scale sizes between MDS-2019-028 and other diplodocids as evidence of small body size, the *Tehuelchesaurus* integument can also be used in favor of this argument since the scale sizes greatly differ depending on where they are located on the body. Del Valle Giménez (2007) inferred that the scales varied in size depending on whether they were ventrally or dorsally located; scale impressions associated with the arms and thoracic region were 1-3mm in diameter, while scales associated with the scapula were 30mm in diameter. MDS-2019-028 has several sections composed of polygonal scales which vary in size depending on whether they are “ventrally” or “dorsally” located. Specifically, polygonal scales from section A2 and B2 are consistently less then 5 mm while polygonal scales in section A3, A4, and most of fragment C are equal to 5 mm. Other than the polygonal scales on MDS-2019-028, there seems to be a general trend in the size of all the scales based on location, since the majority of the small scales are located “ventrally” while all the biggest scales are located “dorsally” (Fig. 13). The high diversity of scales over a small area potentially being indicative of change in body area, combined with the small size of the polygonal scales compared to other Morrison diplodocid polygonal scales, and the similarity in distribution of small and large sized scales like seen in *Tehuelchesaurus*, suggests that MDS-2019-028 belongs to a small individual.

The scales on section B2 curve downward at an extreme angle of 66˚ relative to the square scales closest to the polygonal scales. This type of patterning is often seen on crocodilians, arching around the area where the limbs attach to the body such as the shoulder and waist. Though there is no direct evidence of arching scale rows around the limbs of dinosaurs, it has been noted that in exceptionally well preserved hadrosaur “mummies” such as those of the genus *Edmontosaurus* and *Corythosaurus*, the scales are smallest around the limb regions to accommodate for flexibility (Brown, 1916; Osborn, 1912). It is, therefore, possible that the scales in section B2 may have had the same purpose, and most likely surrounded a limb. As discussed previously, the polygonal scales on the arm of *Tehuelchesaurus* were of similar size to the polygonal scales from the thoracic region (Del Valle Giménez, 2007). The polygonal scales on section B2 are of similar small size as the polygonal scales from section A2, which is consistent with what we currently know of scale size distribution in sauropods if we are to consider section B2 a limb region and section A2 a thoracic region. If true that section B2 belonged to a limb, the limb in question would have been relatively small, considering the shoulder/leg would be no wider than 100 mm (Fig. 2). Despite the evidence, it should be noted that we cannot rule out the possibility that the rib and the skin belonged to the same individual. Although the integument appears to go under the rib on the bedding plane, it is also possible that the integument was originally over the rib but either eroded away or was removed by accident. Additionally, as stated above, the presence of small 1–3 mm scales on *Tehuelchesaurus* bring into question of whether the small scales in MDS-2019-028 indicate small size. It’s possible that the ventral scales of *Diplodocus* were larger in general compared to overall body size to the ventral scales of *Tehuelchesaurus*, or that the small ventral scales in large sauropods are a result of negative allometry. More research is required to understand the full extent of diplodocid integument, and how it may differ in size and distribution in individuals of different sizes. However, we still find the following evidence worth discussing, as it opens discussion on how integument can be used to determine more than just a dinosaur’s appearance, but also to indicate an individual’s size and determining location of the integument on the body without skeletal material present.

## Conclusion

The skin (MDS-2019-028) discovered at the Mother’s Day Quarry shows new scale shapes and orientations never before seen in *Diplodocus* sp., including rectangular, globular, and ovoid scales as well as the arching scale rows orientation. Scale diversity and orientation on this small patch of integument strongly suggests the skin belonged to a very small individual and potentially a “juvenile”. If this can be confirmed, MDS-2019-028 may provide information on the ontogenetic development of diplodocid scales. We would also like to compare MDS-2019-028 to other *Diplodocus* sp. skin fossils from the Mother’s Day Quarry for future studies into diplodocid integument. More research can also be conducted in the taphonomic reasons why diplodocid skin is more commonly preserved as carbonaceous film rather than impressions in the Morrison formation. This discovery also highlights the scientific significance of the Mother’s Day Quarry and the potential to find additional skin fossils during future excavations.

## Additional Information and Declarations

### Field Study Permissions

The following information was supplied relating to field study approvals (i.e., approving body and any reference numbers): The United States Department of the Interior Bureau of Land Management approved fieldwork (permit numbers: MTM 109606, MTM 109606-e1, MTM 109606-e2, MTM 109606-e3, MTM 109606-e4).

### Data Availability

The following information was supplied regarding data availability: The raw data are the figures.
